# Supplementary material for: Post-pandemic face-to-face learning environments for undergraduate students: A scoping review protocol
Source: PLoS One. 2024 Nov 13;19(11):e0309932. doi: 10.1371/journal.pone.0309932 (PMC11559994; doi:10.1371/journal.pone.0309932)
Supplement: S1 Appendix — (DOCX) [file pone.0309932.s002.docx]

Search strategy

| Database: | Dimensions |
| --- | --- |
| Date of search | June 15, 2024 |

| Search | Search criteria (equation) | # Records retrieved |
| --- | --- | --- |
| # 1 | ("Post pandemic" OR "Postpandemic" OR "Postcovid-19" OR "Postcovid" OR "Pospandemia" OR "Post-covid") AND ("University students" OR "Undergraduates") AND ("Face-to-face instruction" OR "In-person instruction" OR "Face-to-face learning" OR "In-person learning") | 5376 |
| # 2 | (“Postpandemic” OR “Postcovid-19”) AND (“Undergraduate” OR “Undergraduate”) “Face-to-Face Learning” | 573 |
| # 3 | “Postpandemia” Y (“Estudiantes Universitarios” O “Pregrado”) Y "Aprendizaje presencial" | 6 |

| Database: | Redalyc |
| --- | --- |
| Date of search | June 15, 2024 |

| Search | Search criteria (equation) | # Records retrieved |
| --- | --- | --- |
| # 1 | ("Post pandemic" OR "Postpandemic" OR "Postcovid-19" OR "Postcovid" OR "Pospandemia" OR "Post-covid") AND ("University students" OR "Undergraduates") AND ("Face-to-face instruction" OR "In-person instruction" OR "Face-to-face learning" OR "In-person learning") | 4 |
| #2 | (“Postpandemic” OR “Postcovid-19”) AND (“Undergraduate” OR “Undergraduate”) “Face-to-Face Learning” | 3 |
