# Supplementary material for: Post-pandemic face-to-face learning environments for undergraduate students: A scoping review protocol
Source: PLoS One. 2024 Nov 13;19(11):e0309932. doi: 10.1371/journal.pone.0309932 (PMC11559994; doi:10.1371/journal.pone.0309932)
Supplement: S2 Appendix — (DOCX) [file pone.0309932.s003.docx]

Appendix II - **Data extraction instrument**

| **Information sources** | | **Name** | **No.** | **Author** | **Country of origin** | **Year of publication** | **URL/DOI** |
| --- | --- | --- | --- | --- | --- | --- | --- |
|  | -* |  |  |  |  |  |  |

* Dropdown list to select the source of information

Datebase, Web page, Gray literature, preprints,

technical notes, Policy reports

(continuation)

| **Aims** | **Population** | **Method (s) - Study design - type of study** | **Researh instruments** |
| --- | --- | --- | --- |
|  |  |  |  |

(continuation)

| **Outcomes** | **Concept (Key Findings)**  Researh question: What is known about the effects of post-Covid-19 pandemic face-to-face learning environments on undergraduate students? | | |
| --- | --- | --- | --- |
|  | What cognitive, emotional, and communicative demands characterize undergraduate students face-to-face learning during the post-pandemic period? | What changes have been made in pedagogical or teaching-learning strategies in face-to-face education after the pandemic? | What are the main recommendations in terms of guidelines, policies and programs related to face- to-face education during the pandemic? |

Source: adapted from Karteczka-Świętek K, et al (2022).
