# Supplementary material for: Post-pandemic face-to-face learning environments for undergraduate students: A scoping review protocol
Source: PLoS One. 2024 Nov 13;19(11):e0309932. doi: 10.1371/journal.pone.0309932 (PMC11559994; doi:10.1371/journal.pone.0309932)
Supplement: Attachment — Submitted filename: En_PONE-D-24-02071.pdf [file pone.0309932.s004.pdf]

PONE-D-24-02071 – Post-pandemic face-to-face learning environments in undergraduate health sciences students: a scoping review protocol

Thank you for inviting me to review this manuscript and provide an opinion.
The authors will examine the important issue of post-pandemic in-person learning environments for health students. To achieve this, they are proposing a scoping review, using the JBI methodology. The methodological approach is appropriate, taking into account the objectives and research question.
Overall, the protocol is well written. I followed the steps proposed by the JBI methodology and articles previously published by PLOS ONE. It was based on these two references that I prepared my opinion.
I suggest some adjustments to the protocol to ensure the reliability and reproducibility of the review.

I answered questions 1 and 2 as partly because the **search strategy** and **data extraction** needs to be clear, as I describe later.

The last paragraph of the introduction describes the preliminary search and is appropriate, as JBI recommends, “The first step is an initial limited search of at least two appropriate online databases relevant to the topic." However, the details of databases, gray literature sites, and reference list searches must be clearly described in the protocol method. This information appears in the abstract, but is not included in the protocol method. The information can be within the search strategy or you can create a specific topic, “Information Sources." See these protocols, for example:
Within the search strategy:
https://journals.plos.org/plosone/article?id=10.1371/journal.pone.0269495

Topic “Information Sources”:
https://journals.plos.org/plosone/article?id=10.1371/journal.pone.0296659

Regarding information sources, it needs to be clear what the databases and gray literature sources will be and, also, add the search on websites of organizations for references on the subject, as appears in the method in the last example mentioned.

The authors chose to collect only in the English and Spanish languages. Which is not recommended by the JBI Manual. The recommendation is that “The time frame (start and end dates) chosen for the search should be clearly justified and any language restrictions specified (e.g. “only sources of evidence published in English were considered for inclusion”)." The authors need to eliminate this criterion or justify your choice. If researchers only speak English and Spanish, this does not justify the exclusion of the others languages. By the way, as it is a post-pandemic study, it must establish and justify what will be considered “post-pandemic”. See: https://jbi-global-wiki.refined.site/space/MANUAL/4687620/11.3.7.1+Search+strategy

The method informs that the evaluation “will be screened by three independent reviewers." Although the manual allows this to be “is performed by two or more reviewers, independently," it is totally acceptable for the evaluation to be carried by two independent reviewers and the third to be the judge in cases of disagreement. This would make a fourth evaluator unnecessary to resolve disagreements. As the team is made up of four people, it is possible to form two pairs of evaluators, and someone from the other pair can be a judge in the case of disagreement. This can make document evaluation faster. This paragraph is just a suggestion.

The extraction instrument needs to be improved, incorporating details of the questions and inclusion criteria that will be collected. For example, the concept appears: “aspects related to cognitive and emotional demands, pedagogical adjustments, detected barriers to participation, and study habits." I suggest that each topic be collected separately; this will make it easier to organize the results. If they collect them all together, the authors will have trouble separating them later. All the data that will be collected must be described, both in the method and in the extraction instrument. This is not clear in the protocol. As it stands, it is unclear what data will be collected.

* In addition to enabling reproducibility, the clearly constructed extraction instrument will facilitate researchers' work. We developed a scope review, in the research group that I lead and left the instrument very open. When it came to organizing the results, it was much more difficult.

Reference 10 is wrong, the correct one is “Peters MDJ, Godfrey C, McInerney P, Munn Z, Tricco AC, Khalil, H. Chapter 11: Scoping Reviews (2020 version). Aromataris E, Munn Z, editors. JBI Manual for Evidence Synthesis. JBI; 2020. Available from https://synthesismanual.jbi.global . https://doi.org/10.46658/JBIMES-20-12

I suggest that the other references be reviewed; I didn't check them all.
