# Supplementary material for: Post-pandemic face-to-face learning environments for undergraduate students: A scoping review protocol
Source: PLoS One. 2024 Nov 13;19(11):e0309932. doi: 10.1371/journal.pone.0309932 (PMC11559994; doi:10.1371/journal.pone.0309932)
Supplement: Attachment — Submitted filename: Response to reviewers.pdf [file pone.0309932.s005.pdf]

Bogota, april 06 - 2024

Dear
Editorial Manager
Plos One

Following the instructions received, we have reviewed the manuscript titled 'Post-pandemic face-to-face learning environments in undergraduate health sciences students: a scoping review protocol.' We are now submitting responses to each of the points raised by both the editor and the reviewers.

## About journal requirements

| |
|---|
| 1. Please ensure that your manuscript meets PLOS ONE's style requirements, including those for file naming. The PLOS ONE style templates can be found at https://journals.plos.org/plosone/s/file?id=wjVg/PLOSOne_formatting_sample_main_body.pdf and https://journals.plos.org/plosone/s/file?id=ba62/PLOSOne_formatting_sample_title_authors_affiliations.pdf |
| **Authors response**<br>Yes, we have made sure to maintain the style requirements |

| |
|---|
| 2. We noticed you have some minor occurrence of overlapping text with the following previous publication(s), which needs to be addressed:<br><br>DOI: 10.11124/JBIES-22-00147<br><br>In your revision ensure you cite all your sources (including your own works), and quote or rephrase any duplicated text outside the methods section. Further consideration is dependent on these concerns being addressed. |
| **Authors response**<br>We have thoroughly reviewed the reference article titled "Barriers and facilitators for implementing a nurse residency program for new graduate nurses: a scoping review protocol", and we identified the similarity with the phrase "The results will be presented in diagrammatic or tabular format with a narrative summary" therefore, we decided to present a new wording of that line as follows: "The search results will be depicted in the flowchart suggested by PRISMA, and in addition, a narrative summary will be included"; the reference has also been cited in the new version of the manuscript. |

3. Thank you for stating the following in your Competing Interests section: "NO authors have competing interests"

Please complete your Competing Interests on the online submission form to state any Competing Interests. If you have no competing interests, please state ""The authors have declared that no competing interests exist."", as detailed online in our guide for authors at http://journals.plos.org/plosone/s/submit-now

This information should be included in your cover letter; we will change the online submission form on your behalf.

**Authors response**
Once we submitted the protocol on January 22, 2024, the downloadable PDF file includes that we refer to "NO authors have competing interests", however, we will review it and we have also included this statement in a new version of the submission letter.

## About reviewers' comments

1. Does the manuscript provide a valid rationale for the proposed study, with clearly identified and justified research questions?

The research question outlined is expected to address a valid academic problem or topic and contribute to the base of knowledge in the field.

Reviewer #1: Yes
Reviewer #2: Partly

**Authors response**
Thanks to feedback, we adjusted the scope of the questions as follows:

**Initial questions in the first submission**

1. What are the effects of the pandemic on face-to-face learning environments in undergraduate health sciences students?
2. What concepts and research methodologies are reported on post-pandemic face-to-face learning environments?

**New questions**
General Question
1. What is known about post-pandemic (Covid-19) face-to-face learning environments for undergraduate students?

Subquestions
1. What are the cognitive, emotional and communicative demands that characterize students in post-pandemic in-face to face learning?
2. What are the changes in pedagogical or teaching-learning strategies in post-pandemic face-to-face education?
3. What are the main recommendations in terms of guidelines, policies or programs related to post-pandemic face-to-face education?

2. Is the protocol technically sound and planned in a manner that will lead to a meaningful outcome and allow testing the stated hypotheses?

The manuscript should describe the methods in sufficient detail to prevent undisclosed flexibility in the experimental procedure or analysis pipeline, including sufficient outcome-neutral conditions (e.g. necessary controls, absence of floor or ceiling effects) to test the proposed hypotheses and a statistical power analysis where applicable. As there may be aspects of the methodology and analysis which can only be refined once the work is undertaken, authors should outline potential assumptions and explicitly describe what aspects of the proposed analyses, if any, are exploratory.

Reviewer #1: Partly
Reviewer #2: Partly

**Authors response**

To enhance clarity, we decided to focus the study on "undergraduate students," regardless of their field of study, and to reformulate the study's general question, as well as to define three sub-questions. In this way, we hope to have improved the data extraction tool to facilitate the analysis and interpretation of the findings.

3. Is the methodology feasible and described in sufficient detail to allow the work to be replicable?

Descriptions of methods and materials in the protocol should be reported in sufficient detail for another researcher to reproduce all experiments and analyses. The protocol should describe the appropriate controls, sample size calculations, and replication needed to ensure that the data are robust and reproducible.

Reviewer #1: Yes
Reviewer #2: Yes

**Authors response**
No comments

4. Have the authors described where all data underlying the findings will be made available when the study is complete?

The PLOS Data policy requires authors to make all data underlying the findings described in their manuscript fully available without restriction, with rare exception, at the time of publication. The data should be provided as part of the manuscript or its supporting information, or deposited to a public repository. For example, in addition to summary statistics, the data points behind means, medians and variance measures should be available. If there are restrictions on publicly sharing data—e.g. participant privacy or use of data from a third party—those must be specified.

Reviewer #1: Yes
Reviewer #2: No

**Authors response**
Inside the manuscript it is indicated that the protocol is available in the Open Science Framework repository, accessible from this link (https://doi.org/10.17605/OSF.IO/6P9QB)

5. Is the manuscript presented in an intelligible fashion and written in standard English?

PLOS ONE does not copyedit accepted manuscripts, so the language in submitted articles must be clear, correct, and unambiguous. Any typographical or grammatical errors should be corrected at revision, so please note any specific errors here.

Reviewer #1: No
Reviewer #2: Yes

**Authors response**
The new version of the manuscript was carefully revised and grammatical meanings were adjusted.

6. Review Comments to the Author

Please use the space provided to explain your answers to the questions above and, if applicable, provide comments about issues authors must address before this protocol can be accepted for publication. You may also include additional comments for the author, including concerns about research or publication ethics.

You may also provide optional suggestions and comments to authors that they might find helpful in planning their study.

(Please upload your review as an attachment if it exceeds 20,000 characters)

Reviewer #1: The scoping review protocol includes important details of the proposed scoping review. The manuscript contains quite a few grammatical issues, which makes some sections difficult to understand. I have pointed out some of these areas, however, did not point out every issue and encourage the authors to carefully review the manuscript for grammatical issues and consider an editing service if needed.

**Abstract**
**Lines 29-30:** The authors may want to consider revising to something such as "This scoping review aims to explore the existing literature related to post pandemic learning environments for undergraduate health sciences students after participating in virtual classes during the COVID-19 pandemic."

> **Authors response**
> The text was adjusted like this:
> "This scoping review aims to explore the existing literature related to post-pandemic face-to-face learning environments for undergraduate students, following their participation in virtual classes during the Covid-19 pandemic".

**Lines 30-31:** Is the secondary aim focused on post-pandemic teaching-learning processes or these processes in general?

> **Authors response**
> According to the reviewers' suggestions, we have decided to highlight specific aspects to guide the review, in order to avoid leaving any findings open-ended, but rather to focus on particular points. Therefore, we have reformulated both the general study question and the secondary objective into 3 sub-questions as follows:
>
> *New Research question:*
> What is known about post-pandemic (Covid-19) face-to-face learning environments for undergraduate students?

*New Research sub questions:*

- What are the cognitive, emotional and communicative demands that characterize students in post-pandemic face to face education?
- What are the changes in pedagogical or teaching-learning strategies in post-pandemic face-to-face education?
- What are the main recommendations in terms of guidelines, policies or programs related to post-pandemic face-to-face education?

**Lines 37-38:** This sentence seems to have some grammatical/punctuation issues.

**Authors response**
Grammatical adjustments were addressed in the new version of the manuscript

**Lines 39-40:** The wording of this sentence is unclear. Is it trying to say that there is little research on this topic?

**Authors response**
Grammatical adjustments were addressed in the new version of the manuscript.

**Line 44:** Is the review looking at studies conducted in 2022 and 2023 or published in 2022 and 2023. It may also be helpful to specify whether this is calendar year or academic year if you're referring to when the studies were conducted.

**Authors response**
For the reader's clarity, the sentence was adjusted like this:

"This review will consider completed and published studies from the calendar years 2022 and 2023 that encompass post-pandemic face-to-face learning environments for undergraduate university students from any geographical área"

**Lines 46-48:** This sentence could benefit from rewording. It would be helpful to put the names of the databases after the term database rather than after the types of publications that will be included.

**Authors response**
The recommendation was addressed and included in the new version of the manuscript.

**Lines 48-49**: It would be helpful to mention the title/abstract screening as well.

**Authors response**
The recommendation was addressed and included in the new version of the manuscript.

**Introduction**
**Line 58**: Change COVID 19 to COVID-19

**Authors response**
The recommendation was addressed and included in the review of the entire manuscript.

**Line 107**: Specify COVID-19 pandemic. The abstract mentions that this is after the return from virtual learning. That may be helpful to specify here as well.

**Authors response**
The recommendation was also addressed for the reformulation of the general research question as follows:

What is known about post-pandemic (Covid-19) face-to-face learning environments for undergraduate students?

**Lines 107-110**: The objectives in the abstract differ somewhat to what is listed here. The wording doesn't need to be identical, but they should be getting across the same points.

**Authors response**
The general objective of the study, as well as the secondary objectives, were adjusted and made corresponding both in the abstract and in the question formulation section within the manuscript.

**Lines 112-131:** The inclusion criteria would fit better under methods rather than introduction. Do the studies included need to specifically address the return to face-to-face learning environments or will studies be included regardless of whether they address this as long as they were conducted in face-to-face learning environments during the timeframe of interest. It would be helpful to specify the time period of the studies you're looking at and indicate whether that time period was when the studies were conducted or published.

**Authors response**

We include inclusion criteria in the framework of the abstract, following the guidelines of JBI_Protocol_Template_Scoping_Reviews. On the other hand, in the new version of the manuscript, we clarify that studies completed or published during 2022 and 2023 will be considered.

**Methods**
**135-136:** "in correspondence" doesn't seem to be the best term to use here

**Authors response**
Grammatical adjustments were addressed in the new version of the manuscript.

**Lines 144-145**: Specify the specific databases that will be searched. These seemed to be mentioned in the abstract, but not the main text.

**Authors response**
Indeed, the databases had been mentioned in the abstract, however, they were also included in the "search strategy" section, in the new version of the manuscript.

**Lines 147:** I suggest saying something like "identify additional relevant papers."

**Authors response**
Yes, this suggestion was included in the corresponding line.

**Line 151**: It would be helpful to specify if this is a pilot test of the title and abstract screening process.

**Authors response**

Yes, right in the same line it is referred to like this: "After a pilot test (20 records), titles and abstracts will be screened…"

**Line 152:** From what's written in the text it sounds like each article will be reviewed by 3 independent reviewers. It would be helpful to add "each" before title and abstract if that's the case.

**Authors response**

Yes, this suggestion was included in the corresponding line.

**Lines 152-153:** It's mentioned that SUMARI will be used for full-text review. Will this also be used for title and abstract screening or will a different program be used?

**Authors response**

Indeed, titles and abstracts will also be included, this clarification was included in the corresponding line.

**Lines 152-153** and **157-158:** I'm a little unclear on the screening process. Do all 3 reviewers need to make the same decision in order for an article to move forward? How is a fourth reviewer going to break a tie between three reviewers – you can get two and two?

**Authors response**

This statement was added to the end of the paragraph: "Any disagreements that arise between reviewers at each stage of the screening process will be resolved by consensus or with a fourth reviewer".

**Line 168**: Change "scope" to "scoping"

**Authors response**

Yes, this suggestion was included in the corresponding line.

**Discussion**

The discussion section would benefit from additional details, including a discussion of the limitations of the protocol.

Search strategy: I encourage the authors to further refine the search strategy and present a draft search strategy for at least one electronic database as outlined in the PRISMA-P checklist the authors included rather than information on the limited preliminary search that only provides one term for each concept.

Reviewer #2: Thank you for inviting me to review this manuscript and provide an opinion.

The authors will examine the important issue of post-pandemic in-person learning environments for health students. To achieve this, they are proposing a scoping review, using the JBI methodology. The methodological approach is appropriate, taking into account the objectives and research question.

Overall, the protocol is well written. I followed the steps proposed by the JBI methodology and articles previously published by PLOS ONE. It was based on these two references that I prepared my opinion.

I suggest some adjustments to the protocol to ensure the reliability and reproducibility of the review.

I answered questions 1 and 2 as partly because the search strategy and data extraction needs to be clear, as I describe later.

**Authors response**

Thank you very much, adjustments were indeed made to the protocol with the recommendations of the two reviewers.

The last paragraph of the introduction describes the preliminary search and is appropriate, as JBI recommends, "The first step is an initial limited search of at least two appropriate online databases relevant to the topic." However, the details of databases, gray literature sites, and reference list searches must be clearly described in the protocol method. This information appears in the abstract, but is not included in the protocol method. The information can be within the search strategy or you can create a specific topic, "Information Sources." See these protocols, for example:

Within the search strategy: https://journals.plos.org/plosone/article?id=10.1371/journal.pone.0269495
Topic “Information Sources”: https://journals.plos.org/plosone/article?id=10.1371/journal.pone.0296659
Regarding information sources, it needs to be clear what the databases and gray literature sources will be and, also, add the search on websites of organizations for references on the subject, as appears in the method in the last example mentioned.

**Authors response**

In the new version of the manuscript, in addition to databases, we include websites of international organizations that present reports on education, such as The UNESCO International Institute for Higher Education in Latin America and the Caribbean (IESALC), and the Inter-American Development Bank, and regarding grey literature or preprints, we mention SocArXiv.

The authors chose to collect only in the English and Spanish languages. Which is not recommended by the JBI Manual. The recommendation is that “The time frame (start and end dates) chosen for the search should be clearly justified and any language restrictions specified (e.g. “only sources of evidence published in English were considered for inclusion”)." The authors need to eliminate this criterion or justify your choice. If researchers only speak English and Spanish, this does not justify the exclusion of the others languages. By the way, as it is a post-pandemic study, it must establish and justify what will be considered “post-pandemic”. See: https://jbi-global-wiki.refined.site/space/MANUAL/4687620/11.3.7.1+Search+strategy

**Authors response**

In the new version of the manuscript, in the “search strategy” section, the reason for using English and Spanish in addition to English and Spanish is justified, as the team's linguistic capabilities and because we do not have translation services for other languages. We have also expanded the discussion section, where we expose the focus on the English and Spanish languages as one of the limitations of the study.

Furthermore, we deemed it important to address what is considered post-pandemic. Therefore, an overview was included from the second paragraph of the introduction of the new version of the protocol.

The method informs that the evaluation “will be screened by three independent reviewers." Although the manual allows this to be “is performed by two or more reviewers, independently," it is totally acceptable for the evaluation to be carried by two independent reviewers and the third to be the judge in cases of disagreement. This would make a fourth evaluator unnecessary to resolve disagreements. As the team is made up of four people, it is possible to form two pairs of evaluators, and someone from the other pair can be a judge in the case of disagreement. This can make document evaluation faster. This paragraph is just a suggestion.

**Authors response**

In the new version of the manuscript and also taking into account the comment of reviewer #1, this clarification was included: “Any disagreements that arise between reviewers at each stage of the screening process will be resolved by consensus or with a fourth reviewer”

The extraction instrument needs to be improved, incorporating details of the questions and inclusion criteria that will be collected. For example, the concept appears: “aspects related to cognitive and emotional demands, pedagogical adjustments, detected barriers to participation, and study habits." I suggest that each topic be collected separately; this will make it easier to organize the results. If they collect them all together, the authors will have trouble separating them later. All the data that will be collected must be described, both in the method and in the extraction instrument. This is not clear in the protocol. As it stands, it is unclear what data will be collected.
*In addition to enabling reproducibility, the clearly constructed extraction instrument will facilitate researchers' work. We developed a scope review, in the research group that I lead and left the instrument very open. When it came to organizing the results, it was much more difficult.

| |
|---|
| **Authors response**<br>Indeed, the data extraction instrument was enhanced based on the recommendations of the reviewers, which, on one hand, allowed for: clarifying the main study question and formulating 3 specific sub-questions. The adjustments are visible in Appendix II of the new version of the manuscript. |

.

| |
|---|
| Reference 10 is wrong, the correct one is “Peters MDJ, Godfrey C, McInerney P, Munn Z, Tricco AC, Khalil, H. Chapter 11: Scoping Reviews (2020 version). Aromataris E, Munn Z, editors. JBI Manual for Evidence Synthesis. JBI; 2020. Available from https://synthesismanual.jbi.global . https://doi.org/10.46658/JBIMES-20-12<br>I suggest that the other references be reviewed; I didn't check them all. |
| **Authors response**<br>This reference was adjusted and the others were revised |

We appreciate the work done by the publisher and the reviewers; it has allowed us to gain precision regarding the scope of the manuscript. We hope the second version meets the publication requirements

Karim Garzón-Díaz
Corresponding author.
