# Supplementary material for: Post-pandemic face-to-face learning environments for undergraduate students: A scoping review protocol
Source: PLoS One. 2024 Nov 13;19(11):e0309932. doi: 10.1371/journal.pone.0309932 (PMC11559994; doi:10.1371/journal.pone.0309932)
Supplement: Attachment — Submitted filename: Response to reviewers.docx [file pone.0309932.s006.docx]

Bogota, June 24 - 2024

Dear

Editorial Manager

Plos One

Following the instructions received, we have reviewed the manuscript titled “Post-pandemic face-to-face learning environments for undergraduate: a scoping review protocol”. We are now submitting responses to each of the points raised by both the editor and the reviewers.

| **Comments Reviewer #1** | **Authors response** |
| --- | --- |
| “There are discrepancies in the title. The information on the title page and version without highlights use the title “Post-pandemic face-to-face learning environments in university students: a scoping review protocol” and the version with the highlights uses “Post-pandemic face-to-face learning environments in undergraduate health sciences students: a scoping review protocol.” | In the previous revision, we presented in the "Response to reviewers" letter (pages #2 and 3) our intention to reformulate the study's questions and sub-questions, and we argued for the adjustment of the title accordingly, thus:  “To enhance clarity, we decided to focus the study on "undergraduate students," regardless of their field of study, and to reformulate the study's general question, as well as to define three sub-questions. In this way, we hope to have improved the data extraction tool to facilitate the analysis and interpretation of the findings”.  On the other hand, in the “Revised Manuscript with Track Changes” file, we also note the adjustment to the title (highlighted in blue). |
| Abstract  Lines 26-28: Since the first component includes commas, it would be beneficial to use semi-colons to separate each element. | Adjustments made. In the “Revised Manuscript with Track Changes” version, semi-colons were included. |
| Line: 44: It’s unclear what is meant by “designed for this purpose.” I suggest specifying this refers to conducting scoping reviews. | Adjustments made, In the “Revised Manuscript with Track Changes” version: “This scoping review will follow the JBI methodology to conducting scoping reviews” |
| Lines 50-51: The wording of this sentence doesn’t make it clear that articles will be screened for eligibility criteria. It sounds like all articles retrieved will be included in the study. | The sentence was adjusted. In the “Revised Manuscript with Track Changes” was included “screen”, before “retrieve”. |

| Lines 51-52: This sentence is a little unclear and conflicts with lines 188-189, which state that conflicts will be resolved by consensus OR a fourth reviewer. The wording of this sentence makes it sound like a fourth reviewer will be brought in to moderate the consensus process among the three original reviewers. | In the “Revised Manuscript with Track Changes”, the wording of lines 188-189 was adjusted to be consistent with lines 51-52.  The authors chose only 3 reviewers, and consensus will be used.  New Lines 54-55-202-206. |
| --- | --- |
| Lines 65-66: The wording of this sentence is somewhat awkward. | In the “Revised Manuscript with Track Changes” the wording was improved.  New Line 71-72. |
| Lines 70-71: There seems to be an “and” missing from “education, health, ecology” | In the “Revised Manuscript with Track Changes” the Word “and” was included.  New Line 79 |
| Lines 95-98: These sentences seem to be missing references | In the “Revised Manuscript with Track Changes”, the reference was included.  New Line 111 |
| Lines 129-130: Change “in-face to face learning” to “in face-to-face learning” | In the “Revised Manuscript with Track Changes”, the hyphen was eliminated.  New Line 129 |
| Line 131: Is this referring to changes compared with pre-pandemic? | Yes, it is referring to changes compared with pre-pandemic. |
| Lines 164-166: This section could benefit from reframing. Different terms representing the concepts of interest is something should be considered in developing all search strategies and isn’t unique to this study. Including different terms used to search for different concepts is beneficial and including an appendix or table with the search terms used for the different concepts within the manuscript can be helpful. The post-pandemic period isn’t the only concept within the research question that has numerous terms associated with it and those should be described here as well. | Within the protocol, we make reference to these controlled terms, anticipating that following the content analysis of the reviews, we may uncover evidence enabling the structuring of a specific appendix |
| Line 177: This sentence has grammatical issues. | In the “Revised Manuscript with Track Changes”, the grammatical issues were improved.  New Lines 195-196 |

| Line 216: This includes grammatical issues | In the “Revised Manuscript with Track Changes”, the grammatical issues were improved.  New Lines 237-238 |
| --- | --- |
| Lines 221-222: The phrase” This will allow, on one hand, to gain a closer approximation to the evidence and diversification of sources” is unclear. | According to this observation and considering the comment from reviewer #2 to not restrict the search to English and Spanish, we decided to adopt this recommendation.  New Lines 245-246 |
| Search strategy: The search strategy provided is a preliminary search and does not reflect the updates to the research question. It focuses on health sciences students. JBI recommends including a full search strategy for at least one electronic database as part of the protocol. | The search strategy is updated based on the controlled terms. |

| **Comments Reviewer #2** | **Authors response** |
| --- | --- |
| The search strategy is presented in Appendix II, but it seems to me to be incomplete. The archive needs to present the complete strategy for databases, which differ in some aspects, for gray literature organizations and websites.  In the search strategy, separate what are the databases, organizations, and websites of the gray literature. This way, it will be more suitable for insertion in the PRISMA flow diagram. | The search strategy presented in Appendix II was adjusted to differentiate between databases and other search sources such as grey literature organizations and websites, among others. |
| Although the PLOS ONE journal itself publishes scoping review protocols that use only English as an inclusion criterion, I maintain the recommendation of not restricting languages for inclusion in the review. The 2024 version of the JBI manual strongly recommends that there be no language restrictions. “Reviewers should include the languages that will be considered for inclusion in the review as well as the timeframe, with an appropriate and clear justification for choices. Our strong recommendation is that there are no restrictions on source inclusion by language unless there are clear reasons for language restrictions (such as for feasibility reasons)”.  The justification that “as the team's linguistic capabilities and because we do not have translation services for other languages” is not reasonable. I clarify that, in my opinion, the issue of language restrictions was not considered in the minor revision recommendation and will not be considered in the next revision of this protocol, but I leave it on record here. | In accordance with this reviewer's observation and the 2024 version of the JBI manual, the author team will adopt the no language restrictions. This adjustment is made in the new version of the manuscript (“Revised Manuscript with Track Changes”).  A new reference was included in this section, citing the new JBI manual.  Additionally, the review timeframe was also adjusted to June 05, 2024 |
| Regarding the selection of studies, the authors maintain that “each of the titles and abstracts will be screened by three independent reviewers for evaluation against the inclusion criteria for review” and add that “any disagreements that arise between reviewers at each stage of the screening process will be resolved by consensus or with a fourth reviewer." It makes no sense. An additional reader is required in the absence of consensus on document inclusion or exclusion. In the case of three reviewers, there will always be a consensus; the three can agree to include or exclude, or there will be two in favor or two against. Are authors considering consensus only when all three readers agree among themselves? I understand that the JBI Manual proposes that the third reviewer is precisely to resolve the lack of consensus. | Following the recommendations, the involvement of a fourth reviewer was removed from the manuscript, and it is maintained that in the event of any discrepancies, consensus will be utilized.  This adjustment is made in the new version of the manuscript (“Revised Manuscript with Track Changes”). |
| Although the authors cited the extracted data will include specific details about the “participants, concept, context, study methods, and key findings relevant to the review question(s) (see Appendix II)”. I did not find the Appendix in the document II. | The following supporting documents are attached: Checklist, Search strategy, and Data extraction instrument. |

We appreciate the work done by the publisher and the reviewers; it has allowed us to gain precision regarding the scope of the manuscript. We hope the second version meets the publication requirements. We also conducted a comprehensive review of the manuscript's grammar and adjusted for the new version.

Karim Garzón-Díaz

Corresponding author.
